# Editorial: Targeting TNF/TNFR signaling pathways

**DOI:** 10.3389/fphar.2022.1120954

**Published:** 2023-01-04

**Authors:** Liqiang Pan, James J. Chou, Tianmin Fu

**Affiliations:** ^1^ Zhejiang University, Hangzhou, China; ^2^ Harvard Medical School, Boston, MA, United States; ^3^ Comprehensive Cancer Center, The Ohio State University, Columbus, OH, United States

**Keywords:** TNFR super family, TNF signaling pathway, cancer immunotherapies, agonistic antibody, death receptor (DR5), fibroblast growth factor (FGF)-inducible 14 (Fn14), FcγRIIB cross-linking

Members of the tumor necrosis factor receptor superfamily (TNFRSF) can be specifically activated to induce death of some cancer cells or to stimulate proliferation of immune cell. There are genuine needs for in-depth understanding of the mechanism by which these receptors are activated, as many of them are targets for immunotherapy (Ashkenazi, 2008; Wajant, 2015).

Past 3 decades of research of TNFRSF have painted a vastly complicated picture of this important family of cytokine receptors both in signaling pathway and in the fundamental mechanism of receptor triggering. It is now clear that activation of these receptors by antibodies is not as simple as concentrating the receptors in the cell membrane by multivalent engagement of the receptor ectodomains, as there appear to be specific conformational and clustering arrangement of the receptors that strongly influence signaling and the mode of ligand-induced receptor clustering can vary significantly between different members of the TNFRSF (Vanamee and Faustman, 2018; Pan et al., 2019). Furthermore, different members of the TNFRSF may have different mechanism of autoinhibition in the absence of ligand.

Hence, therapeutic targeting of TNFRSF signaling pathways needs to be tailored to each member of the receptor family, and this would require in-depth understanding of the various mechanism of receptor autoinhibition and activation adopted by each of the TNFRSF members. The aim of the current Research Topic is to assemble known data and discuss potential strategies for therapeutic targeting of the receptors in the TNFRSF as well as the latest findings about the receptor mechanism. Below are short summaries of the contributions in this Research Topic.

Zaitseva et al. provided a thorough update of targeting fibroblast growth factor (FGF)-inducible 14 (Fn14, also known as TWEAK receptor) for tumor therapy. Fn14 is a small member of the TNFRSF of 14 kDa with its ectodomain consisting of only one cysteine rich domain (CRD). The TWEAK-Fn14 axis has been linked to several physiological processes. In particular, it seems to play an important, beneficial role in tissue repair following acute injury. As the authors suggested, the tissue damage that is unavoidably associated with tumor growth triggers tissue repair and thus the tumor microenvironment contains a variety of Fn14-inducing factors and expression of Fn14 has been accordingly reported for a large variety of solid tumors. The signaling pathway of Fn14 is, however, not straightforward. The authors provide an in-depth analysis of how TWEAK/Fn14-mediated sequestration of TRAF2-cIAP1/2 complexes can enhance TNF-induced necroptosis as well as enhance nuclear translocation of p52-containing NF-κB in the non-canonical NF-κB pathway. In addition, the authors provide insights into how different formats of the ligand TWEAK, soluble and membrane-anchored, can lead to different signaling outcomes. Overall, this comprehensive review article should be very helpful to those interested in developing new strategies to target the TWEAK-Fn14 axis for tumor therapy.

Liu et al contributed an insightful review on antibody-targeted TNFRSF activation for cancer immunotherapy, focusing on the role of Fc-gamma receptor IIB (FcγRIIB) engagement in TNFRSF clustering mediated by agonistic antibodies. TNFR/TNF family interactions provide diversified signals to various types of immune cells at different stages, including inflammation, apoptosis, proliferation and differentiation. Members of the TNFRSF such as CD27, GITR, CD137 and OX40 have been demonstrated to function as T cell co-stimulators. Hence, exploiting the clinical benefit of TNFRSF agonistic antibodies for cancer immunotherapy is of great biomedical interest. The authors reviewed the essential role of FcγRIIB in facilitating multimerization and activation of receptors of the TNFRSF for certain agonistic antibodies, such as DR2, DR5, CD137, OX40, GITR and CD40 agonists. The authors also proposed the working model of xLinkAb for FcγRIIB-dependent TNFRSF clustering bridged by IgGs, which eventually lead to the formation of the multivalent TNFRSFs-IgGs-FcγRIIBs complexes that can activate downstream signaling. Based on the understanding of the structural and functional correlations of agonistic antibodies, the authors pointed out that several Fc-engineering approaches should be extensively evaluated in the context of proper Fab, the epitope, and hinge selection, for optimizing the anti-tumor efficacy while minimizing systemic toxicity.

Zhou et al. discussed the roles of TNF signaling pathways in metabolism of bone tumors. The authors reviewed the effects of TNF superfamily member 2 (TNFSF2), also known as tumor necrosis factor alpha (TNF-α), on bone tumors in terms of metabolic homeostasis. TNF-α has been identified as an important regulator in improving osteoclast formation and inhibiting osteoblast activity. The authors explain that endogenous TNF-α in the tumor microenvironment is associated with inflammatory disorders, giving rise to the bone tumor invasion and progression. Following that, TNF-α enhances αvβ3 integrin expression *via* the MEK/ERK/NF-κB signaling pathway to promote chondrosarcoma cell metastasis. The authors suggest that targeting TNF signaling pathways may provide potential therapeutic benefits for bone tumors treatment.

Dong et al. reviewed the alterations in bone fracture healing associated with TNFRSF signaling. The authors summarized the progress regarding how TNFRs contribute to the process of bone fracture healing. Specifically, the article discussed the roles of three TNFR signaling pathways, including that of TNFR1, TNFR2, and RANK, in different stages of bone fracture healing. TNFR1 is involved in osteoclastogenesis. TNFR2 is associated with osteogenic differentiation. RANK is linked to bone remodeling. The authors summarized the molecular mechanism of these signaling pathways in bone fracture healing and discussed potential therapeutic implications of the above TNFRSF members in bone fracture healing.

Ren et al. provided an extensive review of the structure and function of the death receptor 6. Death receptor 6 (DR6) is a member of the TNFRSF; it is a receptor of the β-amyloid precursor protein (APP) and can induce apoptotic cell death upon ligand binding. Similar to other TNFRSF members, DR6 is a type I transmembrane protein comprising an ectodomain with four cysteine-rich domains (CRDs), a transmembrane domain (TMD), and a cytosolic death domain (DD). The CRDs of DR6 are responsible for binding APP. In this review, the authors summarize the progresses of structural studies on DR6 CRDs, TMD, and DD, as well as the complex of CRDs and APP. Based on the available data, the authors proposed a model of DR6 activation and signaling. Furthermore, the authors discussed the roles of DR6 in different diseases.

The TNF/TNFR signaling pathways can be leveraged for apoptosis-inducing antitumor therapy (e.g., DR5, DR6, Fn14), targeted antitumor therapy (e.g., BCMA), blocking-based immunomodulation (e.g., TNF, RNAKL) or activation-based immunotherapy (e.g., 4-1BB, OX40). During the TNFR downstream signaling, receptor clustering plays an important role from all aspects. Receptor clustering facilitates signal amplification and transduction, which is one of the basic principles of agonistic antibody-mediated TNFR activation, e.g., the essential role of FcγRIIB in multimerizing immunoreceptors of TNFRSF. Furthermore, receptor endocytosis after its clustering facilitates the efficient entry of antibody/ligand-drug conjugates into cytoplasmic lysosome, where toxins could be released intracellularly. The presence of pre-ligand assembly domain (PLAD) and autoinhibition status of certain TNFRSF members suggests novel mechanistic super agonism could be achieved *via* breaking autoinhibition status of extracellular domain with agonistic antibodies targeting unique epitopes of TNF receptors.
